# Health practices and neighborhood experiences among young adults living alone under housing poverty in Seoul, South Korea

**DOI:** 10.1093/heapro/daaf231

**Published:** 2025-12-29

**Authors:** Jihyun Lee, Seunghyun Yoo

**Affiliations:** Independent Researcher, Seoul, South Korea; Department of Public Health Sciences, Graduate School of Public Health, Seoul National University, 1 Gwanak-ro, Gwanak-gu, Seoul 08826, South Korea; Institute of Health and Environment, Seoul National University, 1 Gwanak-ro, Gwanak-gu, Seoul 08826, South Korea

**Keywords:** housing poverty, young adults living alone, health practices, neighborhood environment, health equity

## Abstract

Housing poverty increasingly exposes young adults to insecure and inadequate living conditions that undermine health and well-being. This study examined how young adults living alone under housing poverty in Seoul define and practice health in daily life, adapt to inadequate housing, and interpret their neighborhood environments in relation to well-being. A qualitative exploratory design was employed, using in-depth interviews with 44 participants aged 19–39 who met policy-based criteria for housing poverty in South Korea. Interviews explored daily routines, understandings of healthy living, the use of home and community spaces, and neighborhood perceptions. Data were analyzed using reflexive thematic analysis. Participants viewed deliberate efforts to manage their health as a way to restore order and maintain control in their lives. Managing meals, sleep, and self-care enabled continuity and resilience, though often at the cost of social interaction. However, these practices could not fully counter constraints of inadequate housing, which disrupted rest, limited privacy, and constrained opportunities to recharge at home. To compensate, participants extended activities such as studying, exercising, or resting into community and commercial spaces, which they regarded more as necessary extensions of daily life than leisure options. Despite these adaptations, neighborhoods were often perceived as temporary and emotionally distant, offering functionality but little sense of belonging. These findings highlight housing poverty as a lived social determinant that restricts autonomy, emotional balance, and social participation. Addressing housing insecurity as a health promotion issue requires place-sensitive approaches that reduce psychological burdens while supporting young adults’ everyday health and well-being.

Contribution to Health Promotion

- Reveals that health practices are shaped by housing conditions, not just personal choices.
- Highlights how stress, isolation, and emotional burden can result from maintaining health under housing poverty.
- Shows how maintaining daily routines related to health enable young adults to preserve a sense of stability and control in their everyday lives despite poor and unstable housing conditions.
- Explains how young people adapt neighborhood spaces to support their well-being when home environments fall short.

## Background

Housing is widely recognized as a core social determinant of health (World Health Organization [WHO] 2021). Secure, affordable, and adequate housing conditions affect protection from physical risk, the capacity to sustain routines, the maintenance of social relationships, and psychological well-being (Krieger and Higgins 2002, Cummins *et al*. 2007). In rapidly urbanizing contexts where housing costs have outpaced wages, housing has become a pressing concern for equity and health promotion.

Young adults face particular vulnerability. Rising rents, stagnant wages, and declining access to homeownership have disproportionately exposed younger generations to unaffordable, insecure, or inadequate housing [Arundel and Ronald 2021, Organisation for Economic Co-operation and Development (OECD) 2021]. In many global cities, these dynamics force young people to reside in overcrowded or substandard dwellings or toward peripheral neighborhoods distant from work and social networks (McKinnon *et al*. 2023). Seoul exemplifies this trend in the South Korea (hereinafter “Korea”), where soaring housing prices, insecure employment, and shifting household structures have increased the number of young adults living alone in precarious dwellings (Lally 2022). High youth unemployment and widespread job insecurity intensify economic vulnerability, intersecting with rising housing costs and limiting access to stable housing. Although public rental housing schemes exist, eligibility is based on income and assets rather than employment status, leaving unemployed or economically inactive youth at heightened risk of deprivation (Seoul Institute 2022). National statistics indicate that 64% of householders aged 19–39 in Seoul live alone, and more than half spend over 30% of their income on housing (Statistics Korea 2023). Furthermore, 62.7% of young single-person households are classified as being in asset poverty, underscoring their structural vulnerability (Byeon *et al*. 2023).

Definitions of housing poverty vary across contexts. Internationally, it is measured by affordability, overcrowding, and adequacy [United Nations Committee on Economic Social and Cultural Rights (UN CESCR) 1991, OECD 2021]. The UN CESCR further notes that adequate housing includes security of tenure, habitability, access to services, and suitable location, reflecting broader social and environmental dimensions (UN CESCR 1991 ). In Korea, housing poverty is officially defined for policy and statistical purposes by two criteria: cost burden (spending more than 30% of income on housing) and/or spatial deprivation [living in <14 m^2^ (∼150 ft^2^) per person, according to the government’s minimum housing standards, which require basic facilities such as a bathroom and a kitchenette] (Seoul Institute, 2021). These thresholds support consistent monitoring and policy implementation while also revealing structural housing deprivation, highlighting the gap between rising housing costs and limited affordable supply.

While homelessness represents the most visible and extreme form of housing deprivation, many young adults experience housing insecurity in less overt but structurally related ways. In 2024, Korea had 8008 homeless individuals, 16.8% sleeping rough and 83.2% residing in facilities with young adults comprising about 5% of facility residents (Korea Institute for Health and Social Affairs 2024). Although youth homelessness remains relatively limited, it highlights that homelessness and housing poverty, both shaped by unaffordable housing and labor precarity, manifest as different forms of vulnerability. Recognizing these varied expressions of housing insecurity is important because each shapes daily opportunities for stability, recovery, and well-being.

Research has linked housing poverty to acute and chronic health problems, including infections, injuries, and respiratory illness, as well as long-term risks, including diabetes, obesity, depression, social isolation, and substance use (Krieger and Higgins 2002, Bentley *et al*. 2011, Gibson *et al*. 2011, Ding *et al*. 2024). Housing poverty has also been associated with constraints on health behaviors, including irregular eating, insufficient physical activity, smoking, and heavy drinking (Gibson *et al*. 2011, Kim 2018). Neighborhood environments such as green space, walkability, transportation, and social cohesion shape health behaviors and psychological well-being (Bonnefoy 2007, Wong *et al*. 2018, Stephens *et al*. 2020). Safe public spaces, access to healthy food, and neighborhood trust and cohesion are essential for maintaining health and quality of life (Van Hecke *et al*. 2016). These findings suggest that housing poverty is a structural condition that undermines resilience and limits opportunities for participation (Greenop and Alvarez 2022, Listerborn 2023).

Although housing and health have been widely studied, most emphasized health outcomes and structural determinants, leaving less is known about how young adults interpret and manage health in daily life. Housing is the central context where routines of eating, sleeping, working, and self-care are negotiated under constraints. Understanding how these practices are shaped by housing conditions is therefore critical for public health.

This study addresses a gap by examining how young adults living alone under housing poverty perceive and navigate everyday life through health practices, spatial strategies, and emotional interpretations of place. By approaching housing as a lived social determinant of health, this study highlights the enabling and disabling features of environments that are central to advancing health equity.

This inquiry is guided by four interrelated research questions:

1. How do young adults living alone under housing poverty in Seoul define and practice health to maintain continuity, well-being, and control in their everyday lives?
2. How does housing poverty shape the everyday lives and practices of young adults?
3. What spaces support daily routines and well-being and how are these spaces used or adapted?
4. How do they perceive their neighborhood environments, and how do these perceptions influence their well-being?

## Methods

This qualitative exploratory study employed in-depth interviews with young adults living alone and experiencing housing poverty in Seoul. The interviews explored their everyday health practices, spatial experiences, and perceptions of neighborhood environments to understand how housing poverty shapes health as experienced across physical, social, and emotional dimensions.

### Study setting

This study was conducted in a district of Seoul with the highest concentration of young single-person households experiencing housing poverty. The area covers about 4.9% of Seoul’s total land area, 57.3% of all households were single-person households, with adults aged 19–39 forming the majority (Korean Statistical Information Service 2023, Gwanak-district Office 2024).

The area contains a high density of low-cost, substandard housing. Over half of the residential buildings are multifamily structures, including semibasement and small one-room units. These units are concentrated in steeply sloped neighborhoods, where irregular terrain contributes to spatial isolation and poor housing conditions. Owing to its relatively affordable rent, the district remains a key settlement area for economically vulnerable young adults.

Although the district is well connected to major business and commercial hubs through subway and bus networks, access to transportation and essential services remains uneven, particularly in peripheral and elevated areas. Community centers, green spaces, and health-related facilities are present, but their uneven distribution and limited accessibility contribute to persistent spatial inequality. These conditions make the district a critical site for examining how housing poverty is experienced and navigated by young adults in dense urban settings.

### Sampling and data collection

Participants were recruited using purposive sampling based on predefined criteria, including age (19–39 years) and employment status used as an indicator of life-course stage commonly observed among young adults in Seoul (employed, job seeker, and student). Eligibility criteria included residence of at least 6 months in a Seoul district with a high rate of housing poverty and independent living as a one-person household for at least 2 years, while cohabiting couples and individuals without access to online devices were excluded. Young adults were defined based on the official definition set by the Seoul Metropolitan Government’s Ordinance on Youth, which designates 19–39 years as the youth category (Seoul Metropolitan Government 2015). The definition aligns with the prolonged transition to economic and housing independence observed among young adults in Korea (Han *et al*. 2022). Recruitment utilized online and offline channels related to youth and housing issues, including local community website, social media, and neighborhood bulletin boards. These routes were intentionally selected to reach individuals who met the study criteria. A total of 44 participants were recruited.

All interviews were conducted online via Zoom between July 2021 and May 2022 by the first author and a trained research assistant. They alternated as main interviewer and facilitator, and the fist author attended all sessions to ensure consistency. The assistant was not involved in authorship. Prior to each interview, participants completed a brief demographic questionnaire and were informed of the study’s purpose, procedures, and their right to withdraw at any time. This preinterview form included background questions on age, occupation, housing situation, and self-related health, as well as short validated items on depressive symptoms and counseling experience. These questions were not intended as a formal health screening but served to contextualize participants’ living situations and facilitate interpretation of qualitative findings.

Interviews followed a semistructured format covering health practices, daily routines, spatial strategies, and neighborhood perceptions (Table 1). The interview guide was developed to explore how participants understood and practiced health in their everyday lives, how housing and neighborhood contexts shaped these routines, and how they interpreted their living environments. Each session lasted ∼90 minutes and was recorded. Although both video and audio were captured, only the audio was transcribed and used for analysis, in accordance with informed consent. Participants were clearly notified of this during the consent process. Field notes were integrated into the transcripts to enhance contextual understanding. While the interview guide primarily focused on individual and everyday experiences, rather than explicitly addressing policy and institutional issues, participants often discussed broader contextual factors such as housing conditions, neighborhood infrastructure, and social constraints when reflecting on their health and daily life.

**Table 1:** In-depth interview questions.

1. Daily life and routines	Please describe a typical day in your current life. How has your daily routine changed since you first started?
2. Understanding and practicing “healthy living”	What does ‘healthy living’ mean to you in your everyday context? What practices do you currently maintain or try to maintain for your health? What activities would you like to do for your health but cannot? What prevents you? What behaviors do you do that you think are harming your health? Why do you continue them?
3. Health and the use of space	Where do you usually engage in health-related activities? Please describe outdoor and indoor places, including your home, and the types of activities you do in each place. What do these places mean to you? How do they support or hinder your health practices?
4. Neighborhood experiences and perceptions	In what ways does your neighborhood environment affect your health? Please describe aspects of the neighborhood help or hinder your well-being. How might your perception of this neighborhood change if you were to stay here in the long term?

### Data analysis

This study employed reflexive thematic analysis (RTA) to analyze qualitative data, drawing on Braun and Clarke (2021, 2024, 2025). The first author led the data analysis and interpretation with continuous guidance and feedback from the corresponding author who developed and supervised the overall study. In line with the reflexive orientation, the lead researcher brought a theoretically informed perspective rooted in public health and urban health, alongside personal experience with housing insecurity in early adulthood, which sensitized the researcher to participants’ accounts of spatial constraints. This interpretive lens was further shaped by prior participation in a project led by the corresponding author, including repeated field visits to the study area, and experience writing on neighborhood assets of young adults living alone under housing insecurity. Reflexive practices such as analytic memo writing and mind mapping were used throughout the analysis. These positionalities were not bracketed out but critically engaged with throughout the analytic process to enrich interpretation. The corresponding author, who designed and led the research project for this study, contributed as a critical reviewer, drawing on experience working with community residents, activists, and local institutions, as well as involvement in youth housing policy discussions and healthy city collaborations. These positionalities shaped interpretative dialogue and supported theoretical depth.

The analysis followed Braun and Clarke’s (2013, 2021, 2024) six-phase framework in a flexible and iterative manner. Interview recordings and transcripts were repeatedly reviewed with reflective notes for familiarization. Manual coding supported close engagement with meanings related to health practices, daily routines, spatial responses, and neighborhood perceptions. Codes were iteratively reviewed, compared, and organized into broader patterns that informed preliminary themes. For example, codes concerning maintaining order or following planned schedules were revisited during reflexive discussions to explore their shared meaning in relation to stability and control, which contributed to a theme on structural routines under constrained living conditions. Similarly, accounts of using nearby places for multiple purposes or minimizing travel time were compared across participants to understand spatial adaptation to limited housing environments. These examples illustrate how theme development was guided by interpretative reflection rather than mechanical categorization.

Analytic rigor was supported through sustained reflexivity and collaborative dialogue within the research team. Peer debriefings and analytic workshops were conducted not to achieve coding consensus but to challenge assumptions and deepen contextual interpretation. One research assistant, a young adult with lived experience of housing precarity and involvement in youth civic initiatives, contributed to project design and interview development. Their experiential insight informed both data collection and analytic discussions, enhancing reflexive awareness. Consultations with external experts in urban planning and social welfare further situated interpretations within broader sociospatial contexts, consistent with RTA’s interpretivist commitments to transparency and epistemological coherence (Braun and Clarke 2024, 2025).

All interviews were conducted in Korean, and data analysis drew on the original transcripts to preserve the nuances of participants’ meanings. Key excerpts were translated into English by the first author and reviewed by the corresponding author for conceptual clarity. These translations were then back-translated into Korean using an English–Korean translation tool and compared with the original transcripts to identify any discrepancies. Although the manuscript was written in English, analytic interpretations remained grounded in the original Korean data to maintain fidelity to participants’ lived experiences.

### Ethical considerations

The study was approved by the Institutional Review Board of Seoul National University (IRB no. 2106/002-018, 10 June 2021). All participants were fully informed about study’s purpose, procedure, and voluntary nature of the research prior to data collection. Informed consent was obtained from each participant, and they were assured of their right to withdraw at any time without penalty. To protect anonymity and confidentiality, all identifying information was removed or anonymized during transcription and reporting. Interview recordings and transcripts were securely stored and accessed only by the research team.

## Findings

The demographic and housing characteristics of the study participants are summarized in Table 2. A total of 44 participants were included, with 52% identifying as male and 48% as female. Participants’ ages ranged from 23 to 38 years, with 59% in their 20s and 41% in their 30s. Of the total, 46% were employed either full-time or as freelancers, while 41% were seeking employment and 13% were students.

**Table 2:** General characteristics of study participants.

Variables		Count	Percentage (%)
Legal sex	Male	23	52
	Female	21	48
Age	20s	26	59
	30s	18	41
Employment status	Full-time employee	15	35
	Freelancer	5	11
	Job seeker	18	41
	Student	6	13
Housing type	Studio (one-room)	34	77
	Others	10	23
Residential area	>14 m^2^	16	36
	≤14 m^2^	28	64
Rental type	Monthly rent	32	73
	Jeonse^a^	12	27
Average housing cost (USD)	Monthly rent	323 (deposit: 9230)	
	Jeonse deposit	92 300	
Living area	Area A	20	45
	Area B	22	50
	Area C	2	5
Subjective health status	Very good	3	7
	Good	14	32
	Moderate	20	45
	Bad	7	16
	Very bad	—	—
Depression severity (PHQ-9)	None	14	32
	Mild	12	27
	Moderately severe	15	34
	Severe	3	7
Counseling experience	Yes	11	25
	No	33	75
Total		44	100

^a^Jeonse is a unique real estate property rights system in South Korea, where one leases a place with a substantial deposit upfront usually for a year or two instead of paying monthly rent.

Most participants (77%) lived in studio apartments known locally as ‘one-room’ units, which typically include a private bathroom and kitchenette. Notably, 36% lived in housing units smaller than the national minimum standard for single-person households in Korea (14 m^2^), indicating spatial deprivation.

In terms of housing costs, 73% of participants rented on a monthly basis, with an average rent of ∼323 USD and an average deposit of 9230 USD. The remaining 27% lived under Korea’s “Jeonse” system, in which tenants pay a large upfront deposit (averaging 92 300 USD) instead of monthly rent. More than half of the participants were students or job seekers with little to no stable income, suggesting that their earnings were often below national averages for young adults. According to rental housing market reports, the average monthly rent for one-room apartments under 33 m^2^ in Seoul ranges between 500 and 540 USD, reflecting the generally high rental burden in the city (Chosun Biz 2025, Korea JoongAng Daily 2025). In contrast, the average rent reported by participants was considerably lower than the citywide average. This discrepancy indicates that participants were concentrated in the most affordable and often least desirable segments of Seoul’s housing market, highlighting their constrained economic position within one of the region’s most expensive urban housing markets.

Regarding subjective health, 39% of the participants reported being in either good or very good health. Based on PHQ-9 scores, 34% were categorized as having moderately severe depressive symptoms, and 7% were categorized as having severe depressive symptoms. One in four participants (25%) reported prior experience with psychological counseling, suggesting a substantial level of mental health need within this population.

### Navigating structural instability through daily routines with its emotional and social costs

Young adults living alone under housing poverty described persistent instability stemming from precarious employment, fluctuating income, and inadequate housing. Nearly half held jobs, but even full-time employment did not ensure predictable daily routines or financial security. Many participants navigated frequent career transitions, temporary contracts, or job-seeking periods. The financial burden of rent or large deposits, relative to low or inconsistent income, deepened this sense of precarity (see Table 2). Amid this instability, participants sought something they could reliably manage in daily life. Health management emerged as a concrete way to create everyday stability and maintain a workable rhythm. These practices did not resolve structural instability but preserve functional continuity as participants tried to continue study, working, and meeting responsibilities despite precarious housing and uncertain income. Through consistent routines of eating, sleeping, exercising, cleaning, and managing time, they aimed to sustain capacity to function and a sense of control within unstable conditions.

Participants described health not as lofty aspiration but as a basic requirement for enduring daily challenges and maintaining functional stability. They recognized that poor health could easily disrupt their routines and interfere with their ability to work or study. In a context of limited social and institutional support, health was perceived as one of the few aspects of life within their control. Efforts to protect and sustain health were interpreted as proactive strategies to navigate uncertainty and prepare for the future:

*I’ve realized how important it is to eat, sleep, and exercise properly. I’ve been trying to follow a routine that prioritizes these three habits for a while, but only in the last 3–4 years have I truly managed to stick to it. […] I need to balance eating, sleeping, and exercising. These three things help me stay composed and prevent me from becoming overly sensitive. It might sound obvious, but to me, they are the most essential things.* (female, 31, full-time employee)

*I wake up at 5 a.m. and go to the gym. By 7 a.m., I’m back home, take a shower, have a simple breakfast, and head to work. After finishing work at 6 p.m., I usually buy dinner—either takeout or eat out before coming home. Then, from 8 to 9 p.m., I always do laundry, and I clean almost every day as well. Between 10 and 11 p.m., I end all activities and try to go straight to sleep. I first decided on this routine around 2014, but it was around 2016 that it fully took shape. Since then, I’ve followed this exact lifestyle for 5–6 years without much change.* (male, 29, full-time employee)

Maintaining health through structured routines functioned as an internalized coping resource. Effort to manage health through structured and repetitive routines helped participants maintain a sense of order, self-worth, and emotional control when other aspects of life felt unpredictable. Yet pursuit of health and stability through strict daily regimens came with trade-offs, especially for social relationships and emotional well-being. Many participants described following their routines with near rigidity. They treated sleep schedules, solo meals, and daily exercise as nonnegotiable pillars of their lifestyle. These practices were not casual choices but carefully maintained systems to preserve control in uncertain conditions. To avoid emotional stimulation or interruptions, participants often limited social interactions to protect their routines. For some, especially those who moved to Seoul for work or education, meeting friends was emotionally stimulating but ultimately destabilizing. Such interactions often involved drinking or late nights, which participants said disrupted their physical and mental rhythm for days:

*I avoid making frequent plans to meet friends. I usually meet them once a month, typically on a Sunday. If I have plans to meet a friend, I get so excited the day before or even 2 days before that I can’t concentrate on studying. So, I decided to limit it to once a month.* (female, 23, job seeker)

Avoiding social interaction was not only a financial decision but a self-imposed strategy to protect routine and mental focus. Yet, participants acknowledged that this isolation created a sense of loneliness, which was compounded by housing insecurity and uncertain employment:

*I do feel lonely sometimes. However, meeting friends usually leads to drinking, which disrupts my routine. That’s why I think sticking to my routine and maintaining a more structured lifestyle is healthier. I believe that in order to perform well at work, I also need to pay attention to other aspects of life, such as sleep, diet, and exercise.* (male, 30, full-time employee)

These patterns suggest that structured health routines were less about conventional wellness and more about crafting a livable rhythm amid instability. The rigidity of these routines can be understood not as individual inflexibility but as a relational response to environmental precarity. In this sense, health became not only a site of discipline but also of agency, where young adults negotiated fragility and aspiration within the confines of housing poverty.

**Figure 1:**
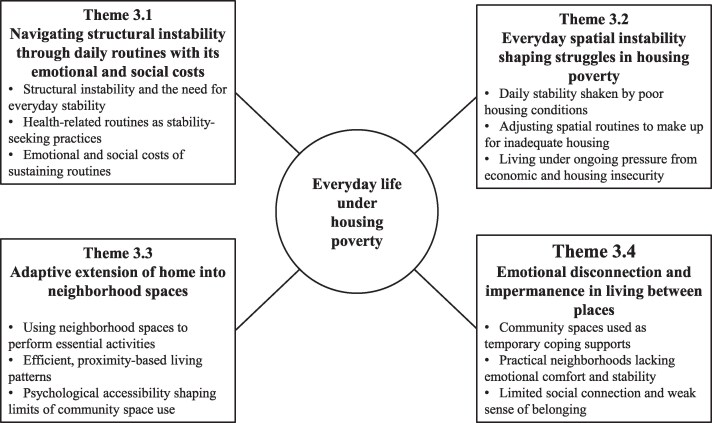
Themes showing how young adult one-person households adapt to daily life under housing poverty.

The analytic findings are organized into four themes (see Fig. 1).

### Everyday spatial instability shaping struggles in housing poverty

While structured routines offered a sense of order and resilience, these efforts were fragile and easily disrupted by material living. Participants faced challenges that made it difficult to sustain even basic routines including cooking, cleaning, resting, maintaining hygiene, and even staying indoors for extended periods. Such disruptions stemmed not only from limited living space and inadequate facilities but also from persistent financial strain and unstable rental arrangements. These constraints shaped daily practices and required continuous negotiation to meet basic needs and maintain well-being.

One immediate challenge was performing basic tasks such as preparing meals, cleaning, or managing personal hygiene. Inadequate kitchen facilities often made cooking at home unfeasible. Many participants relied on takeout or instant food, increasing expenses and undermining health goals. In several cases, the lack of basic household infrastructure, including a sink, stove, or space to store ingredients or a refrigerator, made daily tasks not only inconvenient but also demoralizing:

*Once, I had to wash my dishes in the shower. That moment really made me confront my reality. Water splashed everywhere, and since I had nowhere to place the dishes, I just left them in the shower. After that, I didn’t want to eat in my room anymore.* (female, 36, job seeker)

These accounts illustrate how poor housing conditions go beyond physical discomfort. They compromise a person’s ability to live with dignity and control. While housing poverty in this study was defined by space (<14 m^2^ per person) and/or cost (more than 30% of income), the findings reveal that these dimensions translate into lived experiences of dysfunction and disempowerment. For many, their rooms failed to serve as spaces of rest or recovery, becoming instead sources of ongoing stress and emotional exhaustion.

Spatial limitations also impacted daily maintenance. Drying laundry or cleaning became burdensome in cramped quarters. Many participants described their rooms as too small, dark, or poorly ventilated to spend time in comfortably. In response, they often sought refuge outside, choosing to eat, work, or rest in cafés or public spaces to escape the physical and psychological discomfort of their homes:

*Because of the building in front of mine, sunlight barely comes into my home. It makes staying in my room really uncomfortable. Even when I’m tired during the day, I prefer to go to a café to eat and work, then come home as late as possible.* (female, 24, student)

Changes in spatial practices reflected efforts to adapt daily life to the limitations of poor housing environments. Many participants sought alternative places such as public parks, libraries, or neighborhood sports facilities to support routines difficult to maintain at home. However, access to such spaces was uneven. Some parks were described as too far or poorly maintained, and several participants, especially women, felt unsafe using them during early or late in the day. Distance variation is expected in urban areas, but participants living in low-income housing often reported fewer nearby resources that were both safe and suitable for daily use. The challenge was not only about proximity but also quality or usability of shared spaces. Cafés and other fee-based venues provided comfort but created financial burden for those with limited income. Under these conditions, young adults living alone and experiencing housing poverty struggled to maintain stable routines and care for their well-being within the environments available to them.

Alongside spatial hardship, financial insecurity created pressure. Rising rents, unstable contracts, and frequent relocations made it difficult to form a stable sense of home. Many participants prioritized saving money over investing in food, health, or home improvement, further compromising their quality of life:

*I want to work out to build muscle, but since I’ve been job hunting for a long time, I have to save money. I’d love to eat healthier food, but it’s too expensive, so I just buy the cheapest option. I hope one day I can afford a better lifestyle.* (female, 28, job seeker)

Economic instability and inadequate housing conditions often compelled individuals to compromise their physical and mental health in order to survive. The boundaries of home blurred, shaped not by personal choice but by repeated adaptation to external constraints. Spatial arrangements became improvised and precarious, held together only by uneven support. Housing poverty reshaped how individuals lived, rested, and recovered.

### Adaptive extension of home into neighborhood spaces

Faced with limited living space and ongoing financial insecurity, participants actively expanded their daily routines into the surrounding neighborhoods. Instead of relying solely on their homes, which often lacked comfort, privacy, or adequate basic facilities, they sought out public and commercial spaces to meet basic needs such as rest, study, or stress relief. The local environments served as practical extensions of home, allowing for more flexible and manageable routines.

Commercial indoor venues were especially significant. Local coffee shops and study cafés offered quiet and comfortable environments for studying, working, or spending time. Study cafés, which are hourly rental spaces with private desks, power outlets, stable internet access, and beverages available, were frequently used. These spaces are popular in Korea, especially among students and job seekers who need focused environments. Local coffee shops and study cafés made them affordable and practical alternatives to using one’s own room. Participants also used 24-hour gyms, internet cafés (commonly known in Korea as PC-bangs, primarily used for gaming but occasionally repurposed for study or work), and coin-operated singing booths for late-night stress relief or alternative study spaces. Coin-operated singing booths, soundproof cubicles rented at a low cost, were sometimes used for stress relief or even as private spaces to talk or unwind alone. These spaces provided affordable access without time constraints:

*I usually go to coffee shops to study, read books, or meet people. Sometimes, I play board games with others or spend time enjoying movies and music.* (male, 38, full-time employee)

*To relieve stress, I sometimes go to a singing booth to sing. But during the COVID-19 pandemic, I often had to hold back. When I couldn’t resist anymore, I would go alone. Luckily, there are many such places near my home.* (female, 29, full-time employee)

Many participants also adopted cost-saving strategies when accessing food and other essentials. They frequently visited small diners, convenience stores, and open-air marketplaces that offer affordable meals, groceries, and household goods. These places helped them stretch their limited budgets while meeting basic daily needs:

*I try to save every penny I can, so I buy ‘buy one, get one free’ deals at convenience stores. They are often cheaper than supermarkets.* (female, 36, job seeker)

Although public services technically existed, many participants either did not know about them or were unsure whether those services were truly meant for people like themselves. Some were entirely unaware of youth centers or welfare programs, while others had heard of them as irrelevant or distant. Participants were already overwhelmed by the demands of daily life, and visiting unfamiliar, formal institutions often felt like an extra burden rather than a meaningful opportunity. This reluctance was not simply a result of institutional failure or exclusion, but rather a combination of limited information, low perceived relevance, and emotional distance. The mere existence of programs did not guarantee engagement. For young adults living alone under housing poverty, the lack of accessible, approachable, and clearly targeted outreach often translated into inaction.

Beyond indoor and institutional resources, outdoor public spaces also played an important role in participants’ lives. Proximity to subway stations emerged as a significant locational advantage. These areas offered not only enhanced mobility but also access to well-developed commercial amenities such as affordable gyms, restaurants, and cafés. Participants emphasized that subway access contributed to a sense of autonomy and psychological comfort, particularly because of the safety provided by well-lit, populated streets and the convenience of being able to move easily throughout the city.

*There are many great restaurants near the subway station, making it easy to invite friends over. The station itself is very convenient.* (female, 23, job seeker)

Riverside areas such as ‘Dorimcheon’ were also preferred over mountain trails or more structured, conventional parks. These spaces supported walking, exercise, leisure, and casual socializing. For participants with limited residential space, riversides functioned as accessible everyday environments that promoted physical and emotional well-being:

*I really like Dorimcheon. Whenever I have free time, I frequently go there to exercise. When the weather is nice, it’s the first place that comes to mind. I often see neighbors out for a walk, and the overall atmosphere promotes a sense of health and well-being.* (female, 29, full-time employee)

However, participants who lived in backstreets to save on rent faced limited access to these sources. Poor connectivity to subway stations and commercial areas hindered social interaction and added to their sense of isolation:

*When I feel suffocated during study breaks, I try to take a short walk or go to a coin singing booth with friends. But it’s a bit frustrating because there aren’t many good places to walk nearby—just alleys. Dorimcheon is too far, so I rarely go. […] Right now, transportation is the biggest issue. It’s inconvenient to go anywhere, and I hesitate to invite friends over because of the poor accessibility.* (female, 26, job seeker)

Participants actively repurposed local spaces to meet needs their homes could not fulfill. Yet the presence of neighborhood resources did not guarantee use. Many hesitated to access public services or community spaces, sometimes because they lacked information or worried about cost, but often because these places felt unfamiliar or emotionally distant. Psychological accessibility, meaning whether a space feels approachable, comfortable, and relevant, played a crucial role in shaping use. Even when public centers or youth programs were nearby, participants questioned whether those services were meant for them or felt uneasy entering formal, unfamiliar environments. These emotional and symbolic barriers, though less visible than physical ones, limited their ability to rely on community resources. The findings suggest that well-being depended not just on material availability but on whether environments were perceived as welcoming and personally meaningful. In this context, young adults living alone in housing poverty quietly reconfigured neighborhoods into extensions of home through improvised, emotionally grounded practices of adaptation.

### Emotional disconnection and impermanence in living between places

Although cafés, gyms, and other neighborhood spaces helped ease housing-related challenges, relying on these places as extensions of the home functioned more as temporary coping practices shaped by uneven access, financial limits, and emotional distance. Even in neighborhoods with basic resources, participants found it difficult to feel rooted.

Participants had chosen to live in the low-cost urban neighborhoods located in Seoul’s outer districts. These areas were selected not for long-term residence, but for their affordability and convenience during early stages of independent living. Participants appreciated features such as proximity to subway lines, traditional markets, and fitness centers, which supported their basic needs and routines. Some even noted that they could secure more spacious housing at lower rent compared to central districts:

*Exercise is important to me, so I find it convenient that there are many affordable gyms nearby. Also, commuting within Seoul doesn’t take too long, which reduces my stress. The presence of a traditional market near my home makes it easy to buy ready-made meals. Compared to other parts of Seoul, my living expenses are definitely lower. I think this neighborhood is quite convenient for single-person households.* (male, 28, freelancer)

Despite these practical benefits, participants rarely felt a sense of belonging to their neighborhoods. Their housing choices were driven by economic necessity rather than emotional connection or long-term aspirations. The neighborhoods were often described as physically and psychologically uncomfortable. High-density housing, paper-thin walls, poor ventilation, noise, and lack of greenery made it difficult to rest, concentrate, or feel at ease. These conditions disrupted daily rhythms and produced a persistent sense of instability that permeated both home and neighborhood life:

*When I first moved to Seoul at 29, I chose this neighborhood because it was relatively cheaper compared to other areas. Apartments near subway stations were more expensive and smaller, so I opted for this place where I could get a larger space for the same price, even if it meant climbing a hill. The houses here feel like they share walls like a canvas, so I can hear my neighbor’s sounds very clearly.* (male, 30, full-time employee)

The emotional atmosphere of these areas also contributed to distress. Poorly maintained streets, dim lighting, and the muted presence of others added to feelings of fatigue and detachment. Participants described a heavy mood that shaped not only their impressions of the area but also their self-perception and motivation. Social connections were limited. Narrow alleys, small studio apartments, and frequent tenant turnover left little room for neighborly interaction. Feelings of isolation and invisibility were common, and many longed to leave once they became financially stable:

*When I walk down the street, everyone here looks depressed, and their clothes are dull. Since many are job seekers or students, their clothing often appears plain and lacking in energy. Including myself, I feel like people in this area carry a heavy and dark emotional burden. The atmosphere itself is suffocating.* (male, 25, student)

*I don’t know if it’s because the roads here are in poor condition, but the drainage seems bad, and the streets often feel like a mess. Especially when it rains or snows, it becomes really inconvenient. The neighborhood just feels dirty.* (male, 27, job seeker)

Social disconnection emerged as a persistent challenge. Many participants lived in compact studio apartments where frequent tenant turnover made it difficult to build or maintain relationships. They described feeling isolation and noted that even minimal interactions with neighbors, such as exchanging greetings, were uncommon and often uncomfortable:

*I’ve lived alone for 3 years, and the loneliness is driving me crazy. (…) I don’t think the atmosphere of this neighborhood will change, so I plan to move as soon as I get a job.* (female, 28, job seeker)

Although these neighborhoods met participants’ immediate needs for affordability and access, they seldom fostered a sense of belonging. Participants described them not as homes but as temporary shelters shaped by necessity. Beyond material hardship, they reported a lingering sense of impermanence, living in places that fulfilled basic needs yet lacked emotional stability or long-term direction.

## Discussion

This study qualitatively examined how young adults living alone under housing poverty in Seoul understand and practice health in their daily lives, particularly in relation to the spaces they used and neighborhood environments they inhabit. Previous research has shown that understandings of health vary across social class and broader socioeconomic contexts (D'Houtaud and Field 1984, Nettleton 2020), and our findings add that health was understood as a strategic resource for restoring order and maintaining autonomy amid unstable housing and economic insecurity. Eating, sleeping, exercising, and hygiene were viewed as essential conditions for survival, reflecting the ‘frozen transition’ in which young adults postpone stable adult roles while managing life on their own (Kuhar and Reiter 2012).

Structured routines and spatial adaptations functioned as key survival strategies. These practices helped manage emotional stress and maintain control but also restricted social interaction and intensified isolation. Earlier studies have noted the links between housing poverty, depression, and social withdrawal (Evans *et al*. 2003, Roberts *et al*. 2025), and our findings show that social isolation was shaped as an intentional survival tactic to preserve stability. This highlights how housing poverty constrains choices and can turn self-care into loneliness and anxiety.

This dilemma was shaped by structural limitations rather than individual preference. Deficient housing undermined residents’ ability to rest, cook, and recover at home, pushing to use external spaces such as coffee shops, study cafés, gyms, and riversides for daily routines and emotional recovery. These were experienced as necessary extensions of daily living rather than optional amenities. Access to such spaces was further constrained during the COVID-19 pandemic. Although Korea did not implement a full lockdown, social distancing measures and mobility restrictions limited access to public and commercial spaces, heightening isolation and stress. This period underscored how external disruptions amplify vulnerabilities among those already facing structural housing deprivation and aligns with research emphasizing the role of public spaces in supporting social connection and emotional restoration (Cattell 2001, Peters *et al*. 2010).

Spatial inequalities operated both between young adults experiencing housing poverty and within this group, shaping how participants related to their neighborhoods. Unequal access and use of local resources, affected by financial constraints, distance, and infrastructure gaps, heightened stress and fostered emotional detachment, as many viewed their neighborhoods as temporary and functional rather than places of belonging. Neglected spaces, dim lighting, and disengaged neighbors further limited opportunities to build stability, illustrating that housing poverty is not simply an inadequate shelter but a structural condition that shapes relationships, identity, and possibilities for social regeneration (Nakphong *et al*. 2024).

The findings show that housing poverty affects not just material living conditions but also the ability to manage stress, maintain emotional balance, sustain relationships, and support everyday health. These broader impacts underscore the need to frame housing poverty as a central concern for health promotion and Health in All Policies. Housing insecurity functions as a structural barrier that restricts the daily routines and limits self-determined health practices. In response, young adults develop strategies focused on health management, spatial adaptation, and emotional coping to maintain continuity, autonomy, and psychological stability within prolonged precarity. Yet, these efforts often carry psychological costs, including heightened emotional burden (Kieselbach 2003, Biddle *et al*. 2010, Montserrat *et al*. 2013).

Integrated, place-based health promotion approaches are warranted to align with the everyday realities of young adults experiencing housing poverty. Aligned with recent WHO directions emphasizing the creation of enabling environments to promote health equity (WHO 2023), the Ottawa Charter for Health Promotion (WHO 1986) provides a useful framework for interpreting these findings. The Charter emphasizes creating supportive environments, strengthening community action, and enabling people to regain control over their health, which resonate with participants’ efforts to maintain stability through routines and spatial adaptations. Yet the absence of supportive housing and neighborhood environments highlights the need for local, low-barrier initiatives that can foster emotional security, social participation, and access to health resources.

Building on these principles, effective health promotion interventions should work within the behavioral and spatial patterns that young adults inhabit. Low-barrier, routine-compatible options such as pop-up programs in frequented spaces (e.g. cafés and riverside walkways), opportunities for light group participation (e.g. group plogging activities or community gardening), and digitally facilitated forms of engagement can reduce the emotional burden of formal involvement. Informal activities in everyday places such as riverside walkways, cafés, or small neighborhood groups (e.g. trash pick-up groups, running clubs, or casual board game meetups) supported emotional restoration and light social engagement in our study. These examples illustrate how specific everyday places and activities can function as health-enabling environments when aligned with participants’ routines and preferred modes of interaction.

Theoretically, this study contributes a relational perspective on place by showing how housing, routine, and emotional well-being are interconnected. It demonstrates that housing poverty must be understood as a lived social determinant of health shaped through everyday practices and affective relationships with place. This perspective offers a more nuanced understanding of how young adults navigate instability and how urban environments can operate simultaneously as resources, constraints, and sources of emotional meaning.

This study has several limitations. First, the findings are limited to young adults living alone under housing poverty in a single district of Seoul and may not capture the experiences of other urban contexts or neighborhoods with different social and environmental conditions. Future studies should broaden the scope by including communities with varying urban environments and also consider the perspectives of socially withdrawn or isolated young adults. Second, data were collected during the COVID-19 pandemic, which likely shaped participants’ mobility and neighborhood use. These contextual conditions may differ from postpandemic daily life. Third, this study did not identify clear differences in perceptions of health or neighborhood meanings by gender, age, or employment status. Future research should examine these intersecting identities more systematically using larger-scale surveys, multiple case comparisons, or mixed-methods approaches.

Despite these limitations, this study contributes by showing how young adults living under housing poverty navigate health through both homes and neighborhood spaces. By examining housing and everyday environments together, it highlights how place can simultaneously enable and constrain health practices and illustrates the relational and emotional dimensions of place that are critical for advancing health promotion and equity.

## Conclusion

This study explored how young adults living alone under housing poverty in Seoul managed health living and attach meaning to their neighborhoods from a health promotion perspective. Structured routines emerged as vital for maintaining order, yet they carried emotional and social costs. Inadequate housing conditions further undermined stability, disrupting rest, eating, and recovery. In response, participants extended activities usually done at home into neighborhood spaces to sustain routines and well-being. Many also described their neighborhoods as temporary and emotionally distant, reflecting a condition of living between places rather than experiencing them as supportive communities.

Addressing housing poverty as a health promotion issue requires more than providing affordable housing. It calls for reimagining urban environments as health-enabling ecosystems that support emotional balance, social relationships, and everyday resilience. Codesigned policies, infrastructures, and services that reflect the lived experiences of young adults are essential for relevance, equity, and long-term sustainability. By demonstrating how housing and neighborhood life intersect as lived social determinants of health, this study contributes to health promotion research and the need for interventions that are both place sensitive and socially responsive.

## Data Availability

Due to the sensitive nature of the qualitative data and restrictions imposed by the Institutional Review Board, the full transcripts cannot be shared. Deidentified excerpts relevant to the findings are included within the article.
